# 

**Published:** 1908-11

**Authors:** 


### CONTENTS.

ORIGINAL COMMUNCATIONS—

Long and His Discovery. By I. H. Goss, M. D., Athens, Ga. .......... 401

Should the Appendix be Removed in all Cases Where the Abdomen is Opened for Other Causes? By L. C. Fischer, M. D. .......... 410

Reciprocity Between States. By A. A. Keith, M. D. .......... 415

Some Remarks on Amoebic Dysentery. By Geo. M. Niles, M. D., Atlanta, Ga. .......... 420

The Importance of a Thorough Knowledge of Biology, Bacteriology and the Circulation of the Blood for the Successful Application of serum therapy. By J. C. Grady, M. D., Kenly, N. C 424

A Report of Cases Treated with Ichthyolated Emulsion Compound. By John Roy Williams, M. D., Greensboro, N. C. .......... 430

Serum Therapy in Tuberculosis. By Dr. Louis C. Rouglin, Atlanta, Ga. .......... 434

EDITORIALS .......... 441

NEWS AND NOTES .......... 447

BOOK REVIEWS .......... 451

MEDICAL ITEMS .......... 454

